# Microtubule Assists Actomyosin to Regulate Cell Nuclear Mechanics and Chromatin Accessibility

**DOI:** 10.34133/research.0054

**Published:** 2023-02-21

**Authors:** Jiwen Geng, Zhefeng Kang, Qian Sun, Man Zhang, Peng Wang, Yupei Li, Jiameng Li, Baihai Su, Qiang Wei

**Affiliations:** ^1^ Department of Nephrology, West China Hospital, Sichuan University, Chengdu, 610041, China.; ^2^ College of Polymer Science and Engineering, College of Biomedical Engineering, State Key Laboratory of Polymer Materials and Engineering Sichuan University, Chengdu, 610065, China.; ^3^ Department of Cardiovascular Surgery, West China Hospital, Sichuan University, Chengdu, 610041, China.

## Abstract

Cellular behaviors and functions can be regulated by mechanical cues from microenvironments, which are transmitted to nucleus through the physical connections of cytoskeletons in the cells. How these physical connections determine transcriptional activity were not clearly known. The actomyosin, which generates intracellular traction force, has been recognized to control the nuclear morphology. Here, we have revealed that microtubule, the stiffest cytoskeleton, is also involved in the process of nuclear morphology alteration. The microtubule negatively regulates the actomyosin-induced nuclear invaginations but not the nuclear wrinkles. Moreover, these nuclear shape changes are proven to mediate the chromatin remodeling, which essentially mediates cell gene expression and phenotype determination. The actomyosin disruption leads to the loss of chromatin accessibility, which can be partly recovered by microtubule interference through nuclear shape control. This finding answers the question of how mechanical cues regulate chromatin accessibility and cell behaviors. It also provides new insights into cell mechanotransduction and nuclear mechanics.

## Introduction

Microenvironments are fundamental and important to cells [1]. The mechanical and physical properties of the microenvironments are well known to be sensed by the tissue cells [2] and influence cellular behaviors [3], such as proliferation [4], differentiation [5], death [6], and extracellular matrix synthesis [4]. For example, at the cellular level, mesenchymal stem cells (MSCs) appeared with osteogenic lineage predominating at the environmental modulus higher than 40 kPa and with adipogenesis occurring predominantly at lower than 3 kPa [7]. It is widely known that the cell behaviors are essentially determined by gene transcription. The environmental factors, including the mechanical cues, should finally alter cell transcriptional activity for controlling cell behaviors and functions. The mechanical signals are recognized to convert into biochemical signals through mechanotransduction [8] and further activate the mechanosensitive transcriptional regulators, Yes-associated protein (YAP)/transcriptional coactivator with PDZ-binding motif (TAZ), which translocate from cytoplasm to nucleus under high levels of mechanical stimuli [1,9]. As the transcriptional regulators, YAP/TAZ are definitely involved in the process of gene expression. However, whether the direct force transmission in cells, as initiated by the mechanical signals [10], regulates gene transcription is still unclear.

Microenvironment and nucleus are directly connected by several essential physical connections, including cytoskeleton, nucleoskeleton, and linker of the nucleoskeleton and cytoskeleton (LINC) complex [11]. Increasing substrate stiffness promotes actomyosin stress fiber assembling, which generates intracellular traction force to shape the nuclear morphology and transmits the traction to chromatin through LINC complex and nucleoskeleton. As a result, nucleus could maintain smooth on stiff substrates, while it becomes irregular on softer substrates [12–14]. The actomyosin skeleton, especially the actin cap, is recognized as the key regulator of nuclear morphology and mechanics [15,16], which directly associate with chromatin remodeling and transcriptional activity [17]. The chromatin remodeling is the basis for gene transcription, which provides binding sites for transcriptional factors to target to the genes.

Actomyosin, microtubule, and intermediate filament are the 3 main types of cytoskeletons [18,19]. Among these 3 cytoskeletons, microtubule is the stiffest [20,21], although the actomyosin generates traction force and determines most of the elastic modulus of cells. Vimentin, the most abundant intermediate filament, contributes to cell elastic behavior [22,23]. Since the nucleus is the stiffest organelle, it is reasonable to speculate that its morphology would be influenced by the microtubule. In fact, some previous studies have confirmed that the microtubule is related to the morphology of nucleus [24–26] and even gene expression [24]. It has been observed that the pressure of the microtubule organizing center deformed the nucleus to a crescent shape, when the cells lacked mechanical sensing [12]. However, few studies focused on how mechanical cues were sensed by cell nucleus and regulated gene activity. Whether microtubule is involved in the process of nuclear mechanosensing is unknown yet. The effect of this process on chromatin remodeling, gene expression, and cell behaviors is also needed to be understood.

This study aims to reveal the process of mechanical signals regulating cell transcriptional activity through the cytoskeleton networks. Specifically, the impact of microtubule, especially the actomyosin–microtubule crosstalk, on nuclear morphology and mechanics as well as chromatin accessibility and cellular behaviors is explored. It demonstrates that the microtubule remodeling is the downstream of actomyosin-exerting force. The actomyosin skeleton synergizes with the microtubule to regulate nuclear mechanics. The microtubule is recognized as an indispensable regulator for controlling nuclear morphology, which further mediates the transcriptional activity through chromatin condensation. Most surprising is that microtubule disruption can not only recover the nuclear morphology change but also the loss of chromatin accessibility and the expression of the cell force-downstream genes caused by the reduction of actomyosin-based traction force. Our work adds an important piece in understanding how mechanical signals regulate cellular gene activity.

## Results

### Actomyosin regulates nuclear invagination through a microtubule-dependent way

**Fig. 1.**
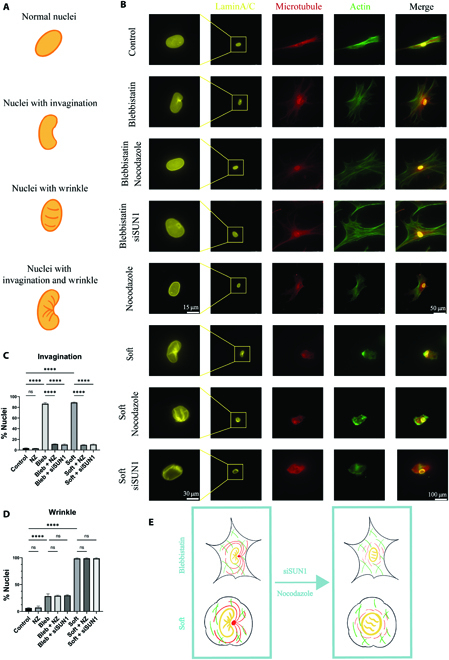
Actomyosin and microtubule synergize to regulate nuclear invagination. (A) Scheme of nuclear invagination and wrinkle. (B) Representative immunofluorescence images for lamin A/C, microtubule, and filamentous actin (F-actin) of the cells treated in shown conditions. (C and D) The percentage of the invaginated and wrinkle nucleus, respectively (*n* = 50, 3 technical replicates). (E) Scheme of the microtubule disruption for regulating nuclear morphology after actomyosin inhibition. The green represents actomyosin, the red represents microtubule, and the yellow represents nuclei. When the actomyosin-based traction force was inhibited by either blebbistatin or soft hydrogels, obvious invaginations and wrinkles appeared on the nuclei. After disrupting microtubule or disconnecting the link between microtubule and nucleus, the nuclear invagination, but not the wrinkle, got recovered. ns, not significant.

To study the association between actomyosin and microtubule cytoskeletons, we firstly tune the intracellular traction force of MSCs by blebbistatin or soft hydrogels (ca. 1 kPa). Since the myosin II is the core force generator in cells, blebbistatin, the selective inhibitor of nonmuscle myosin II adenosine triphosphatases, can efficiently limit cell traction force [27]. The soft hydrogels alter the force balance at the cell adhesive interface to limit the increase of cell traction force [10]. When the cell traction force was inhibited by either blebbistatin or soft hydrogels, there were obvious invaginations and wrinkles on the nuclei (Fig. 1B to D). Please note that the invagination is defined by detecting the negative curvature at the outer edge of the nucleus, while the wrinkle indicates the wrinkle structures observed on the upper surface of the nucleus (Fig. 1A). As the cells spread on the 2-dimensional surfaces and the nuclei are in an anisotropic pancake shape instead of an isotropic spherical shape, the invagination and wrinkle are 2 types of distinct patterns. Figure 1C and D shows the percentage of invaginated and wrinkle nucleus, respectively. As both blebbistatin and soft hydrogel could decrease the intracellular traction force, most of the cells contained invaginated nucleus in either condition (Fig. 1C). The nucleus also wrinkled in the cells on soft hydrogels but not the cells treated by blebbistatin (Fig. 1D). Meanwhile, the nuclear morphology change was not observed when the microtubule was disrupted by nocodazole. It indicated that the actomyosin skeleton played a dominant role in the regulation of nuclear morphology and was enough to maintain the nuclear shape. For comparison, dimethylsulfoxide, the solvent used to dissolve blebbistatin or other reagents, did not affect cell states in the experimental conditions (Fig. S1). In addition, the microtubules gathered inward to the perinuclear area of the blebbistatin-treated cells and the cells on soft hydrogels comparing with the untreated cells on the rigid glass surfaces (control group). The live-cell imaging of actomyosin and microtubule confirmed this phenomenon (Movies S1 to S3 and Fig. S2). The actin filaments were gradually disordered after adding blebbistatin, which was accompanied by the microtubules gathering to the perinuclear area. The gathered microtubules may cause nuclear invagination, since it is a rigid network [12,22].

Therefore, the microtubules were disrupted by nocodazole, the inhibitor of microtubule polymerization. The microtubule disruption did not affect the shape of the nuclei on the rigid glass surfaces without blebbistatin treatment, but, interestingly, recovered the invaginated nuclei of the cells treated by blebbistatin or on the soft hydrogels (Fig. 1B and C). However, the wrinkle pattern of the nuclei on the soft hydrogels cannot be recovered by nocodazole treatment (Fig. 1B and D). The nucleus is connected to the microtubules through Sad1 and UNC84 domain containing 1 (SUN1) protein in the LINC complex [19]. The SUN1 was knocked down (Fig. S4) to investigate whether the nuclear invagination is caused by the extrusion of the microtubule network or the linkage between the nucleus and the microtubules. The SUN1 knockdown did not decrease the microtubule gather. However, it also recovered nuclear invagination but not the wrinkles, like microtubule disruption (Fig. 1B to D). In addition, the nuclear volume and area (but not height) were decreased with the appearance of nuclear invagination and wrinkle and partly recovered by nocodazole treatment or SUN1 absence (Fig. S3).

The results above suggested that the actomyosin skeleton regulates nuclear invagination through a microtubule-dependent way, which requires the linkage between the microtubule network and the nucleus instead of the extrusion exerted by microtubule gather (Fig. 1E). The nuclear wrinkles, which cannot be affected by microtubules, observed on the soft hydrogels but not in the cells treated by blebbistatin. This difference may be caused by the different levels of actomyosin disruption. The blebbistatin thoroughly inhibited the myosin II activity. The wrinkle pattern will be focused on in the next section.

Moreover, the nuclear deformation and recover were accompanied with the nucleoskeleton lamin A/C disordering and smoothening. It has been proven in previous studies that the nuclear shape is determined by nuclear lamina, and lamin A/C are its main structural components. The nuclear lamina directly bears the cytoskeleton force through the LINC complex [28]. It is reasonable to predict that the actomyosin and microtubule skeletons regulate nuclear deformation through lamin A/C.

### Actomyosin regulates nuclear wrinkle through a microtubule-independent way

**Fig. 2.**
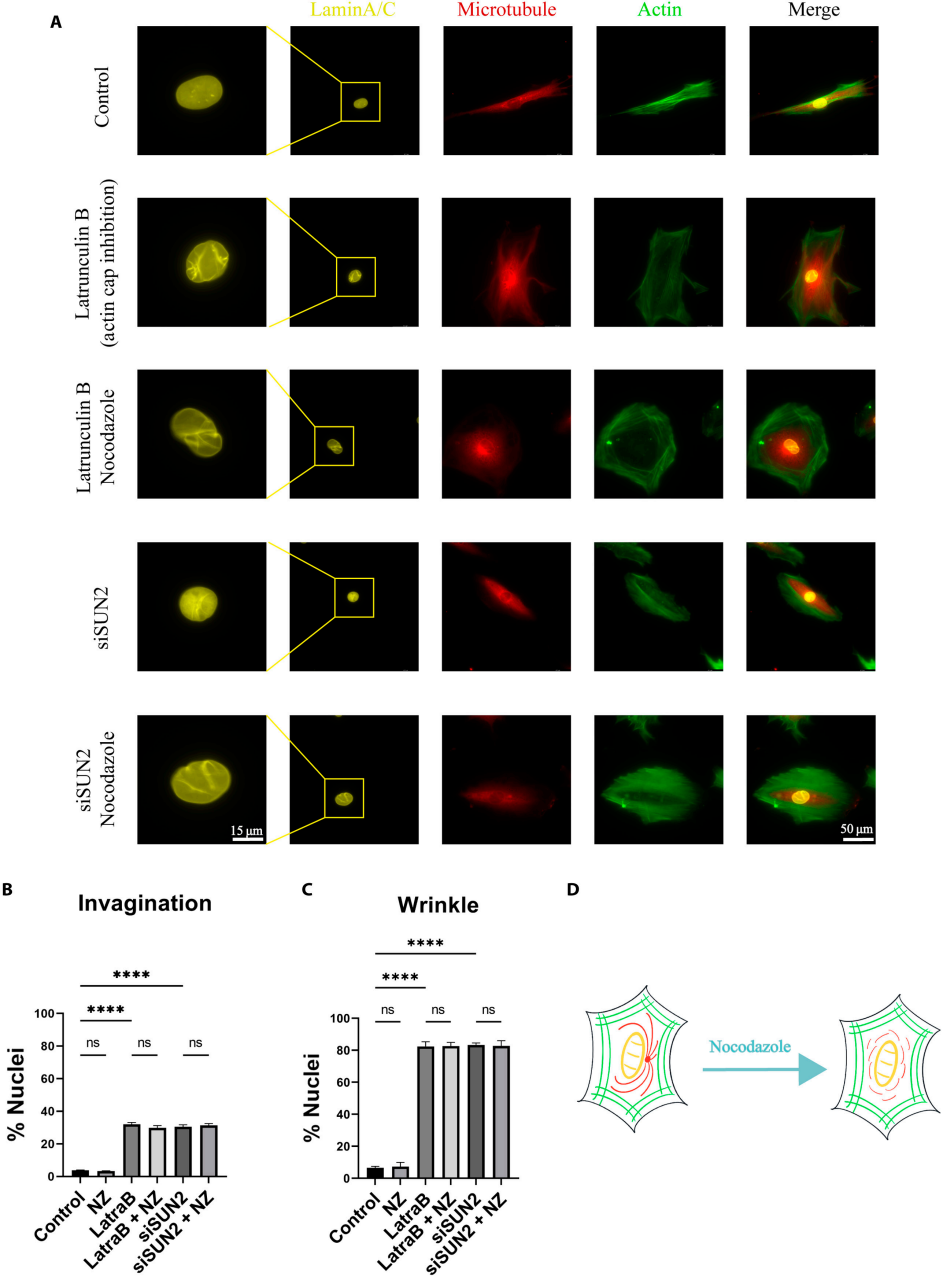
Actomyosin but not microtubule regulates nuclear wrinkle. (A) Representative immunofluorescence images for lamin A/C, microtubule, and F-actin of the cells treated in shown conditions. (B and C) The percentage of the invaginated and wrinkle nucleus, respectively (*n* = 50, 3 technical replicates). (D) Scheme of the microtubule disruption for regulating nuclear morphology after actomyosin–nucleus linkage disruption. The green represents actomyosin, the red represents microtubule, and the yellow represents nuclei. When the actomyosin–nucleus linkage was inhibited, the wrinkles appeared on the nuclear surface independent of microtubule disruption.

To further explore the association among actomyosin, microtubule, and nuclear deformation, we disconnected the link between actomyosin and nucleus but maintained the integrity of the actomyosin network by 2 methods for the cells on rigid glass surfaces. First, low concentration of latrunculin B was utilized to inhibit the actin cap without disrupting the main actomyosin network [16]. Actin cap transmits the actomyosin traction force into nucleus and maintains the nuclear morphology [29]. The other way is knocking down SUN2 (Fig. S4), the linker between actomyosin skeleton and nucleus in the LINC complex [19], The absence of SUN2 also affects the actin cap formation (Fig. 2A). The main difference is SUN2 knockdown can avoid the influence of actomyosin on the nucleus without affecting actomyosin itself. Although low concentration of latrunculin B does not disrupt the main actomyosin network, it still impacts actin polymerization in a certain degree.

With actin cap inhibition, obvious wrinkles appeared on about 85% of the nuclei. Similar to the cells on soft hydrogels, nocodazole cannot recover the wrinkles on the nuclear surfaces (Fig. 2A and C). The SUN2 knockdown experiments confirmed the importance of the connection between actomyosin skeleton and the nucleus, as almost the same results were obtained as the actin cap inhibition. The SUN2 absence resulted in clear nuclear wrinkles independent of nocodazole treatment. In addition, both actin cap inhibition and SUN2 absence led to the microtubules gathering toward the perinuclear area where the actin cap was missing. Due to the presence of the relatively complete actomyosin network, the gathered microtubules only slightly invaginated about 40% of the nuclei (Fig. 2B), which was much lower and weaker than disrupting the whole actomyosin network by blebbistatin. The invagination here was more closed to the wrinkles and could not be recovered after adding nocodazole. Furthermore, the nuclear volume and area were also decreased by wrinkles, which was independent of nocodazole treatment or SUN2 absence (Fig. S3).

These results suggested that the actomyosin skeleton regulates nuclear wrinkle through a microtubule-independent way. The actomyosin skeleton stretches the nucleus to transmit the traction force, which increases the nuclear mechanics and results in the smooth nuclear surfaces. The lack of actomyosin–nucleus linkage, e.g. actin cap inhibition or SUN2 absence, breaks the traction force transmission, although the actomyosin network almost maintains its integrity (Fig. 2D). Thus, the nucleus only bears the pressure from the actomyosin fibers. The wrinkles appeared just under the actomyosin fibers and match their impressions in many nuclei with actin cap inhibition or SUN2 absence (Fig. S5).

### Effects of actomyosin and microtubule disruption on intracellular traction force

**Fig. 3.**
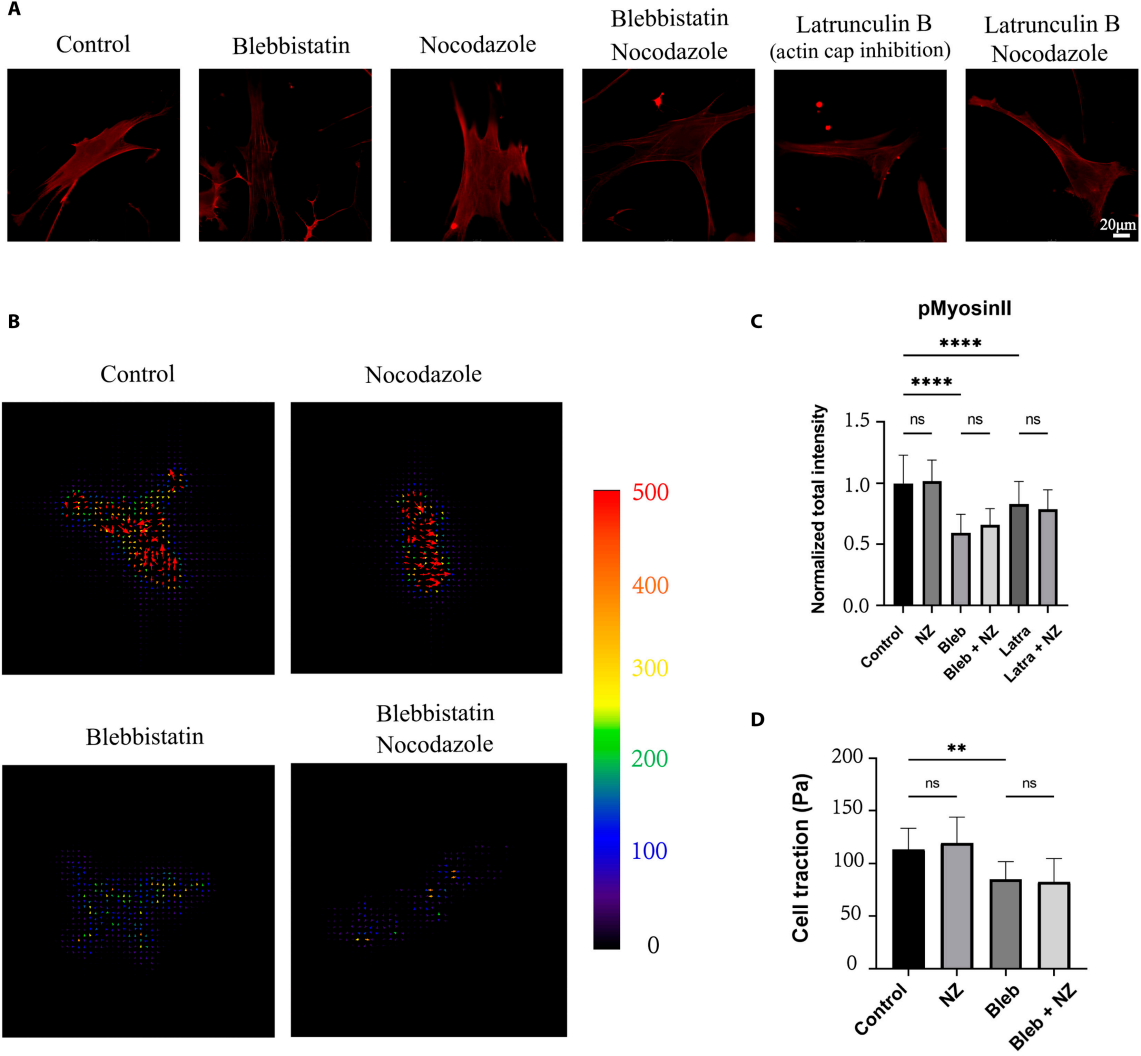
Intracellular traction force. (A) Representative immunofluorescence images and the (B) fluorescent intensity for the phosphorylation of myosin IIa at Ser1943 of the cells treated in shown conditions (*n* = 50, 3 technical replicates). (C) The representative traction fields and (D) the mean traction force of a single cell treated in shown conditions on the hydrogels with embedded fluorescent beads (*n* = 15, 3 technical replicates).

The nuclear morphology is mediated by intracellular traction force [30], and the nuclear deformation has been proven above to be synergistically regulated by actomyosin and microtubule. To figure out whether this process was related to the change of cell traction force, the phosphorylation level of myosin IIa at Ser1943 was monitored, which mirrored the intracellular traction force [31,32]. There was no doubt that blebbistatin, the myosin II inhibitor, efficiently decreased the phosphorylation level of myosin IIa (Fig. 3A and B). The low concentration of latrunculin B decreased myosin IIa activity as well, as latrunculin B inhibited actin polymerization to a certain level. It is not surprising that the lack of intracellular force or the break of force transmission deforms the nucleus. The decrease of the traction force after blebbistatin treatment was confirmed by traction force microscopy (TFM) as well (Fig. 3C and D). The microtubule disruption by nocodazole changed neither myosin IIa activity nor traction force (Fig. 3). Therefore, the recovered nuclear invagination resulting from microtubule disruption is not induced by increasing myosin II-based intracellular traction force, although the inhibition of microtubule formation can activate guanine nucleotide exchange factor to promote actin polymerization [33]. Here, the traction force is more sensitive to actomyosin skeleton and less affected by microtubule networks. The microtubule gather itself regulates nuclear invagination through the microtubule–nucleus connection.

### Actomyosin and microtubule synergize to regulate chromatin accessibility

**Fig. 4.**
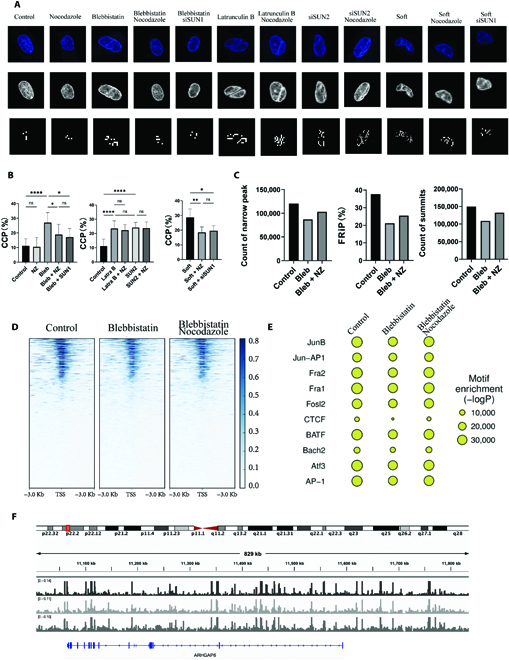
Cytoskeleton-induced nuclear deformation mediates chromatin accessibility. (A) Representative heatmaps of the DAPI intensity and the chromatin condensation parameter (CCP) maps estimated by corresponding edge detection method. (B) The average CCP of the cells (n = 15, 3 technical replicates). (C) The summary of the ATAC-seq results of the cells as indicated by the count of narrow peak, the fraction of reads in peaks (FRiP), and the count of summits per group. (D) The chromatin accessibility over the transcription start site (TSS) of the cells. (E) The TF motif enrichment of the cells. (F) The chromatin accessibility for *ARHGAP6* in the cells with different treatment.

The nuclear morphology is directly related to the transcription activity and cell behaviors [17]. We thus investigated the effects of nuclear invagination and wrinkle on chromatin condensation, the basic premise of gene transcription. Chromatin condensation parameter (CCP) was used to quantitatively analyze the degree of chromatin condensation. The value of CCP is negatively correlated with the chromatin accessibility. As the results show, the change of CCP was related to nuclear morphology (Fig. 4A and B). Both nuclear invagination and wrinkle caused CCP increasing, i.e., more chromatin closed and was not accessible for gene transcription. It is in line with the previous studies that the transmission of the intracellular force in nucleus is required to open chromatin. The interruption of the factors for force generation and transmission, such as actomyosin assembly, actin cap formation, actomyosin–nucleus connection, etc. increases CCP [34,35]. Here, all treatments that led to nuclear deformation increased CCP, including blebbistatin, soft hydrogel, low concentration of latrunculin B, and SUN2 knockdown. Nocodazole alone did not affect CCP, so it was not necessary to test SUN1 knockdown alone. However, the nocodazole treatment or SUN1 knockdown induced nuclear recover after invagination could partly recover the CCP. The wrinkle induced by low concentration of latrunculin B or SUN2 knockdown cannot be recovered, and thus, the related CCP maintained a high level after nocodazole treatment.

The global chromatin accessibility of the invaginated and recovered nuclei was further investigated by assay for transposase-accessible chromatin using sequencing (ATAC-seq). The control group of the cells on glass surfaces, the blebbistatin-treated cells, and the blebbistatin-plus-nocodazole-treated cells were analyzed to verify the regulatory effects of the microtubule on chromatin condensation and accessibility. Compared with the control group, genome in the blebbistatin-treated group became less accessible, while the chromatin accessibility increased after adding nocodazole, as indicated by the fraction of reads in peaks, the count of narrow peak, and the count of summits per group in Fig. 4C. The chromatin accessibility over the transcription start site and the transcription factor (TF) motif enrichment were shown in Fig. 4D and E. The transcriptional activity exhibited the similar trend to the chromatin accessibility, which was decreased in the blebbistatin-treated group and partly recovered in the group of blebbistatin-plus-nocodazole treatment. For instance, *ARHGAP6*, coding for Rho GTPase Activating Protein 6, is important in mechanotransduction and a downstream gene of cell force sensing [36]. As shown in Fig. 4F, the chromatin accessibility for *ARHGAP6* was lost in the blebbistatin group as the intracellular traction force was limited. The nocodazole recovered the invaginated nuclei, thus also recovering the accessibility for *ARHGAP6*.

In sum, both types of nuclear deformation, i.e., invagination and wrinkle, inhibited chromatin accessibility via increasing the chromatin condensation level, which was induced by the disordered nucleoskeleton lamin A/C as discussed above. The microtubule disruption recovered nuclear invagination and nucleoskeleton assembly to reopen chromatin for transcriptional accessibility.

### Microtubule regulates the early markers of the force-downstream cell behaviors

Since the microtubule disruption recovered chromatin accessibility from the deformed nuclei, we are interested in the regulation of intracellular-force-downstream cell behaviors. The MSC osteogenic differentiation as well as cell apoptosis and proliferation are well known to be regulated by cell force sensing [9]. The MSC osteogenic differentiation requires intracellular traction force to shuttle YAP/TAZ into the nucleus and to increase chromatin accessibility for YAP/TAZ target genes. The health-adherent cells can only proliferate in the presence of intracellular traction force, as the force disruption initiates the apoptosis process [9].

**Fig. 5.**
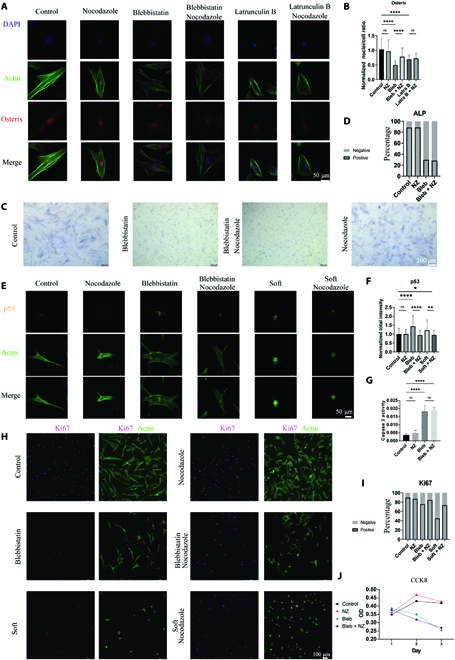
Microtubule regulates the early markers of cell osteogenic differentiation, apoptosis, and proliferation. The ALP staining was performed after cell culturing for 7 d in growth media with shown treatment. Other immuno-fluorescent images were acquired after cell culturing for 1 d. (A) Representative immunofluorescence images and the (B) fluorescent intensity for osterix of the cells treated in shown conditions. (C) Representative images for ALP staining and (D) the percentage of ALP-positive cells after cell culturing for 7 d in growth media with shown treatment. (E) Representative immunofluorescence images and (F) the fluorescent intensity for p53 of the cells treated in shown conditions. (G) The activity of apoptotic downstream enzyme caspase 3 of the cells treated in shown conditions. (H) Representative immunofluorescence images for Ki67 of the cells treated in shown conditions (enlarged images are shown in Fig. S6). (I) The percentage of Ki67-positive cells treated in shown conditions. (J) The cell counting kit 8 (CCK8) analysis of the cells treated in shown conditions. Each statistical result includes more than 50 cells, 3 technical replicates.

Osterix is a TF for the osteogenic differentiation and is recognized as the early marker of osteogenesis. The decreasing intracellular force caused by blebbistatin or low concentration of latrunculin B reduced the nucleocytoplasmic ratio of osterix (Fig. 5A and B). The nocodazole itself did not affect the cytoplasmic-nuclear shuttling but partly recovered the nuclear localization of the osterix in the cells treated with blebbistatin. The trends were consistent with the results of CCP and chromatin accessibility. The alkaline phosphatase (ALP), late marker of osteogenesis, was analyzed as well (Fig. 5C and D). Despite the recovery of the early marker, the ALP cannot be affected by microtubule disassembly.

P53 was detected as the upstream regulator of apoptosis [37]. Culturing cells with blebbistatin or on soft hydrogels increased the P53 activity, while adding nocodazole reduced the P53 to the regular level (Fig. 5E and F). However, the activity of apoptotic downstream enzyme, caspase 3 [38], could not be recovered by nocodazole treatment, although it was obviously activated in the cells treated by blebbistatin (Fig. 5G).

Ki67 has been widely accepted as a marker of proliferating cells [39], which represents the opposite cell phenotype to p53. The percentage of Ki67-positive cells decreased, when the cells were cultured with blebbistatin or on the soft hydrogels (Fig. 5H and I). The nocodazole efficiently promoted the Ki67-positive cells from those treatments. Similar to the cell apoptosis, the recovery of the early regulator Ki67 by nocodazole treatment failed to increase cell number or prevent cells from dying (Fig. 5J), as detected by cell counting kit 8 (CCK8).

From the results above, the nuclear morphology recovery induced by microtubule disruption can successfully recover the early markers and regulators of different force-downstream cell behaviors. However, this regulation did not cause the recovery of the final cell phenotypes. It is reasonable that the cell-phenotype determination also requires the actomyosin activity and the related signaling factors. In particular, focal adhesion kinase phosphorylation requires the traction force exerting on the molecular clutches, which would transduce the mechanical signals to a set of signaling pathways for mediating cell phenotypes [28]. The nuclear mechanics and cytoplasmic mechanotransduction synergize to control cell behaviors and functions.

## Discussion

Nucleus is the most important organelle, in which the gene expression takes place. [15,40]. The nuclear mechanics is balanced by diverse types of forces including traction, tension, extrusion, etc., most of which are exerted by the cytoskeleton networks [15]. The cytoskeleton consists of actomyosin, microtubule, and intermediate filament and serves as mechanical elements to mediate cellular shape, structure, and functions [41,42]. Among the 3 kinds of cytoskeleton, actomyosin and microtubule are more dynamic than intermediate filament [18]. Actomyosin mainly generates and transmits traction force [22] while microtubule is the stiffest cytoskeleton to bear various force [20,21]. The crosstalk between actomyosin and microtubule plays an important role in cell shape determination [18]. Although several studies revealed the function of actin or microtubule alone on regulating nuclear morphology and gene expression [24–26], few of them involved their coordination.

**Fig. 6.**
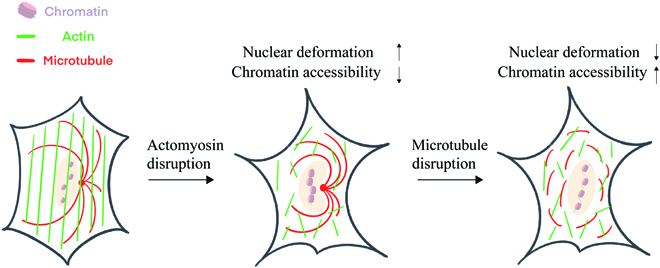
Schematic summary of how actomyosin and microtubule synergistically regulates nuclear deformation and chromatin remodeling. Microtubule negatively regulates the actomyosin-induced nuclear invaginations but not the nuclear wrinkles. The actomyosin disruption leads to the loss of chromatin accessibility, which can be partly recovered by microtubule disruption without affecting cell force.

Our results demonstrated that the actomyosin can regulate nuclear morphology through microtubule-dependent and microtubule-independent ways (Fig. 6). The former mainly caused the nuclear invaginations, while the latter mainly caused the nuclear wrinkles. The intracellular traction force from the actomyosin activates nucleoskeleton lamin A/C to maintain the nuclear shape. The traction force inhibition alters the lamin A/C distribution, which is considered as the reason for nuclear deformation [12,28].

Here, the microtubules are detected to gather toward the nucleus and indent the nucleus through the LINC connection. Microtubule disruption recovers the nuclei from invagination. Therefore, the nuclear invagination should be the result of the combined effects of lamin A/C distribution and microtubule gather, both of which are regulated by actomyosin traction force. Although the association between actomyosin and microtubule has been studied in the past years, the detailed mechanism is still unclear. Previous studies indicate that there must be crosslinker proteins between the microtubules and actin bundles interacting with microtubule plusendbinding proteins [43–46]. A recent study [47] has also reported that the microtubule alters integrin dynamics and affect cell adhesions. As the actin bundles link to the focal adhesions, it provided evidence that there is some coordination between the microtubules and actin filaments. Our results agree with these opinions. When cells are cultured on the soft hydrogels or treated with blebbistatin, a new force balance forms between the disrupted actomyosin networks and microtubules. The microtubule plus-ends lose the pulling force from the actomyosin network and thus move to the direction of minus-ends or microtubule organizing center to indent the nucleus. However, the nuclear invagination is also SUN1-dependent. The SUN1 knockdown recovers nucleus from invagination, although does not change the distribution of microtubule around the nucleus. Therefore, the gathered microtubule may drag instead of press the nucleus for invagination formation.

The disruption of actin cap by low concentrations of latrunculin B or SUN2 knockdown also leads to microtubules gathering toward the perinuclear areas where the actin network is missing. Since the main parts of the actomyosin network are still complete, the microtubules in this case do not indent the nucleus. Actin cap disruption breaks the force transmission into the nucleus, which limits lamin A/C assembly to soften the nucleus [30]. The soft nucleus cannot bear the pressure of the remaining actin stress fibers, which may be the main reason causing wrinkles.

A previous study suggested that gene expression could be altered when actin or microtubule was inhibited alone [24]. How the cytoskeleton networks regulated gene expression was the remaining question. We explored the effects of cytoskeleton-induced nuclear deformation on the chromatin accessibility, which describes the degree that nuclear macromolecules can physically contact the chromatinized DNA [48]. It reveals the way cytoskeleton mediates cellular gene expression.

As the results, both nuclear invagination and wrinkle increase the chromatin condensation level to close the force-downstream genes and switch the force-related cell functions, such as osteogenic differentiation, proliferation, apoptosis, etc. It has been detected for the first time that the microtubule disassembly can partly recover the chromatin accessibility from the invaginated nucleus. Therefore, the activity of some early markers of osteogenic differentiation, proliferation, and apoptosis processes is recovered. It further supports the previous opinion that the chromatin accessibility and transcriptional activity are related to the nuclear morphology [49]. However, the cell phenotypes of these processes cannot be altered by microtubule disassembly due to the lack of intracellular traction force. It agrees with the importance of the intracellular traction force on determining cell behaviors. It would be interesting to reveal at what level the microtubule-associated nuclear mechanical changes can influence the cell behaviors and functions. The differences between nuclear invagination and wrinkle on affecting transcriptional activity and cell functions are also worthy of further exploration.

Microtubule polymerization is central to various biological functions, including cellular motility, vesicular transport, cell division, and so on [50,51]. Microtubule interference has been utilized in tumor therapy to inhibit cancer cell mitosis [52,53]. In fact, more effects and approaches of microtubule treatment can be considered in the advanced technologies for tumor therapy. For example, the microtubule-associated proteins can mediate cellular stress responses [54]. Previous studies have demonstrated that cancer cells have the ability to grow in the absence of actomyosin force [55], while the actomyosin and its force are essential for most of the normal adherent cells [56]. How the cancer cells activate the chromatin accessibility without intracellular traction force is an unsolved question [57]. According to our results, it would be worth paying attention to the relationship between microtubule assembly and nuclear mechanics.

Our study explored the influence of intracellular force transmission on chromatin remodeling. It has been demonstrated that microtubule is involved in the process of force-induced nuclear deformation and contributes to generating invagination in nucleus in the absence of actomyosin force. The nuclear invagination and wrinkle caused by cytoskeleton networks decreased the nuclear volume, leading to closing of chromatin and the loss of chromatin accessibility. However, microtubule interference could alter the nuclear shape change to partially reopen chromatin and switch the transcription activity, which is intimately related to cell functions. This finding complements the pathways of cell mechanosensing and mechanotransduction as well as the understanding of the regulation of mechanical signals on cellular gene transcription. It may be important in understanding the process of development and disease.

## Materials and Methods

### Polyacrylamide hydrogels

Polyacrylamide (PA) hydrogels were prepared according to the protocol in our previous study [7]. Glass coverslips were incubated in the mixed solution of acetic acid, 3-(trimethoxysilyl) propyl methacrylate (Sigma) and ethanol (1/1/14) and then washed 3 times with 96% ethanol. The functionalized coverslips can form covalent attachment of hydrogel substrates to glass. A solution containing 0.05% ammonium persulfate, 0.5% N,N,N′,N′-tetramethylethylenediamine and aqueous suspension of 0.1% 0.5-μm fluorescent beads (only for TFM test) were mixed with acrylamide and crosslinker N,N methylene-bis-acrylamide in different ratios to make hydrogels with different stiffness. The corresponding acrylamide concentration to prepare the 2-, 15-, and 40-kPa hydrogels were 6%, 12%, and 18%, respectively. The corresponding N,N methylene-bis-acrylamide concentration were 0.12%, 0.3%, and 0.6%, respectively. The PA substrates were incubated in N-sulfosuccinimidyl-6-(4′-azido-2′-nitrophenylamino) hexanoate (sulfo-SANPAH, Sigma, 803332), then activated with ultraviolet irradiation for 5 min. After washing with Hepes, PA substrates were incubated in collagen I (ThermoFisher, A1048301) overnight. The stiffness of the PA hydrogels was measured by rheometry. The measurement parameters are as follows, the preload force is 0.2 N, and the frequency is 1 Hz.

### Cell culture and treatment

Human adipose-derived MSCs were cultured in minimum essential medium α (Gibco, 12571063) supplemented with 10% bovine growth serum (Gibco, 10099141C) and 1% penicillin/streptomycin (Gibco, 15140122) at 37 °C with 5% CO_2_. Glass coverslips without hydrogels were also incubated in collagen I (ThermoFisher, A1048301) for 10 min before seeding cells. For inhibitor experiments, drugs and corresponding concentrations are as follows: blebbistatin (50 μM, Sigma, B0560), nocodazole (0.1 μM, Abcam, ab1230630), and latrunculin B (1 μM, Abcam, ab144291). Inhibitors remained in the media during cell culture. Transfections were carried out with Lipofectamine RNAiMax (Invitrogen) for small interfering RNA. The sequences of small interfering RNA used in this study were shown in Table S1. CCK8, caspase-3 assay kit (Colorimetric) (Abcam, ab39401), and ALP stain kit (Solarbio, G1480) were used according to the protocol.

### Immunofluorescence staining and imaging

Cells were washed once with cell culture medium and twice with phosphate-buffered saline (PBS) before fixation with 4% paraformaldehyde at room temperature for 30 min. Samples were then washed 3 times with ice-cold PBS. Cells were permeabilized with 0.25% v/v Triton X-100 in PBS for 10 min at room temperature and then washed 3 times with PBS. Nonspecific antibody binding was blocked by incubating samples with 1% w/v bovine serum albumin (BSA) in PBST (0.1% v/v Triton X-100 in PBS [PBST]) at room temperature for 60 min. Next, samples were washed briefly with PBST and incubated with primary antibodies overnight at 4 °C. Afterward, samples were washed twice with PBST and 3 times with PBS. Samples were then incubated with 4′,6-diamidino-2-phenylindole (DAPI), fluorophore-labeled phalloidin (Thermo Fisher Scientific, A12379) and secondary antibodies, for 60 min at room temperature, followed by washing twice with PBST and 3 times with PBS. Immunofluorescence images were acquired on a Leica DMi8 microscope or a Zeiss LSM900 confocal laser scanning microscope. The spreading area, aspect ratio, average orientation angle, and average relative intensity were all measured by the ImageJ software with at least 50 cells per sample.

The live cell fluorescent probes were the gifts from the group of Prof. Lu Wang. The probes were added to incubate with cells for 1 to 2 h before imaging [58,59]. Live-cell imaging was acquired on a Leica DMi8 microscope for acquiring images every 5 min.

Primary antibodies and corresponding concentrations were used as follows: rat monoclonal anti-Tubulin (Abcam, ab6160, 1:1000), mouse monoclonal anti-Lamin A+C (Novus Biologicals, NB100-74451, 1:200), rabbit monoclonal anti-Osterix (Abcam, ab209484, 1:200), mouse monoclonal anti-p53 (Cell Signaling Technology, #2524, 1:200), rabbit monoclonal anti-Phospho-Myosin IIa (Cell Signaling Technology, #14611, 1:200), and Rabbit polyclonal anti-Ki67 (Abcam, ab15580, 1:200).

Secondary antibodies used were Goat anti-Rabbit Alexa Fluor 568 (Thermo Scientific, 1:500 dilution), Goat anti-Mouse Alexa Fluor 568 (Thermo Scientific, 1:500 dilution), Goat anti-Mouse Alexa Fluor 647 (Thermo Scientific, 1:500 dilution), Goat anti-Rat Alexa Fluor 647 (Thermo Scientific, 1:500 dilution).

### Western blot

Cells were lysed with radio-immunoprecipitation assay lysis buffer (APExBIO, K1020), supplemented with protease inhibitor cocktail (APExBIO, K1007) and phosphatase inhibitor cocktail (APExBIO, K1015). Insoluble cell debris was removed by 15-min centrifugation at 13,500×g at 4 °C. Protein concentration was determined using a bicinchoninic acid protein assay kit (Beyotime, P0010S). Western blot was performed on total protein extracts. Proteins were then transferred to 0.45-μm polyvinylidene difluoride membranes (Sigma, IPVH00010) after electrophoresis. Membranes were blocked for 1 h at room temperature in blocking buffer (Beyotime, P0023B) and incubated overnight at 4 °C with antibodies in primary antibody dilution buffer (Beyotime, P0023A). Goat-anti-Rabbit horseradish peroxidase-conjugated (Abcam, ab2057181:10,000) antibody was used as secondary antibody. Protein bands were visualized with Clarity Western ECL Substrate (Bio-Rad, USA) and the pictures were obtained by ChemiScope Western Blot Imaging System (Clinx Science Instruments, China).

Primary antibodies and corresponding concentrations were used as follows: rabbit monoclonal anti-SUN1 (Abcam, ab124770, 1:1000) and rabbit monoclonal anti-SUN2 (Abcam, ab124916, 1:1000). Glyceraldehyde 3-phosphate dehydrogenase (Abcam, ab8245, 1:1000) was used as a loading control.

### TFM

TFM was conducted according to the protocol in our previous study [7]. PA hydrogels containing fluorescent nanobeads were fabricated as described above. The beads were mixed in the hydrogel precursors and sunk during polymerization due to their own weight. After converting the hydrogels, the micro-beads were kept near the surface of the hydrogels. After coating collagen I, the cells were seeded on the hydrogels. Fluorescent microscopy was used to take images of beads as well as spread cells. Finally, the cells were removed by treating with 1% sodium dodecyl sulfate for 10 min on the microscopy stage. The images were utilized for defining the original position of the dye-labeled beads.

ImageJ plugin “Align slices in stack” was used to correct the experimental drift of the samples. The displacement field in a spread cell region was subsequently calculated by a “particle image velocimetry” plugin in ImageJ. The obtained result was reconstructed using the “Fourier transform traction cytometry” plugin to generate the traction force field as a vector plot.

### CCP

CCP is based on the amount of free space and number of visible edges in DAPI-stained images of the nucleus [60,61]. The condensation of chromatin increases the number of distinct spaces within the nucleus, which is detected by the Sobel edge detection algorithm [62]. The density of edges within the nucleus, normalized to its cross-sectional area, is defined as the CCP [63]. The gradient-based Sobel edge detection algorithm and the estimation of CCP were conducted using MATLAB.

### ATAC-seq

The ATAC-seq and analyses were carried out by Novogene Technology Co., Ltd. ATAC-seq was performed as previously reported [64]. Briefly, nuclei were extracted from treated cell samples, and the nuclei pellet was resuspended in the Tn5 transposase reaction mix. The transposition reaction was incubated at 37 °C for 30 min. Equimolar Adapter 1 and Adatper 2 were added after transposition. polymerase chain reaction was then performed to amplify the library. After the polymerase chain reaction, libraries were purified with the AMPure beads, and library quality were assessed with Qubit.

### Statistics

GraphPad Prism 9 was used for statistical analysis. One-way analysis of variance and post hoc Tukey's multiple comparison test were performed. Significance was demonstrated by *P* < 0.05 (*P < 0.05, **P < 0.01, ***P < 0.001, ****P < 0.0001).

## Data Availability

Data are available from the corresponding authors upon request.
