# Supplementary material for: Microtubule Assists Actomyosin to Regulate Cell Nuclear Mechanics and Chromatin Accessibility
Source: Research (Wash D C). 2023 Feb 21;6:0054. doi: 10.34133/research.0054 (PMC10076026; doi:10.34133/research.0054)
Supplement: Supplementary 1 — Figs. S1 to S6. Table S1. [file research.0054.f1.docx]

**Microtubule Assists Actomyosin to Regulate Cell Nuclear Mechanics and** **Chromatin Accessibility**

Jiwen Geng^a,b,#^, Zhefeng Kang^c,#^, Qian Sun^b^, Man Zhang^b^, Peng Wang^b^, Yupei Li^a^, Jiameng Li^a^, Baihai Su^a,*^, Qiang Wei^b *^

^a^ Department of Nephrology, West China Hospital, Sichuan University, Chengdu, 610041, China

^b^ College of Polymer Science and Engineering, College of Biomedical Engineering, State Key Laboratory of Polymer Materials and Engineering Sichuan University, Chengdu, 610065, China

^c^ Department of Cardiovascular Surgery, West China Hospital, Sichuan University, Chengdu, 610041, China

^#^These authors contributed equally to this paper.

*Corresponding author. Sichuan University, College of Polymer Science and Engineering, State Key Laboratory of Polymer Materials and Engineering, 610065, Chengdu, China. E-mail address: wei@scu.edu.cn.

*Corresponding author. Sichuan University, West China Hospital, Department of Nephrology, 610041, Chengdu, China. E-mail address: subaihai@scu.edu.cn.


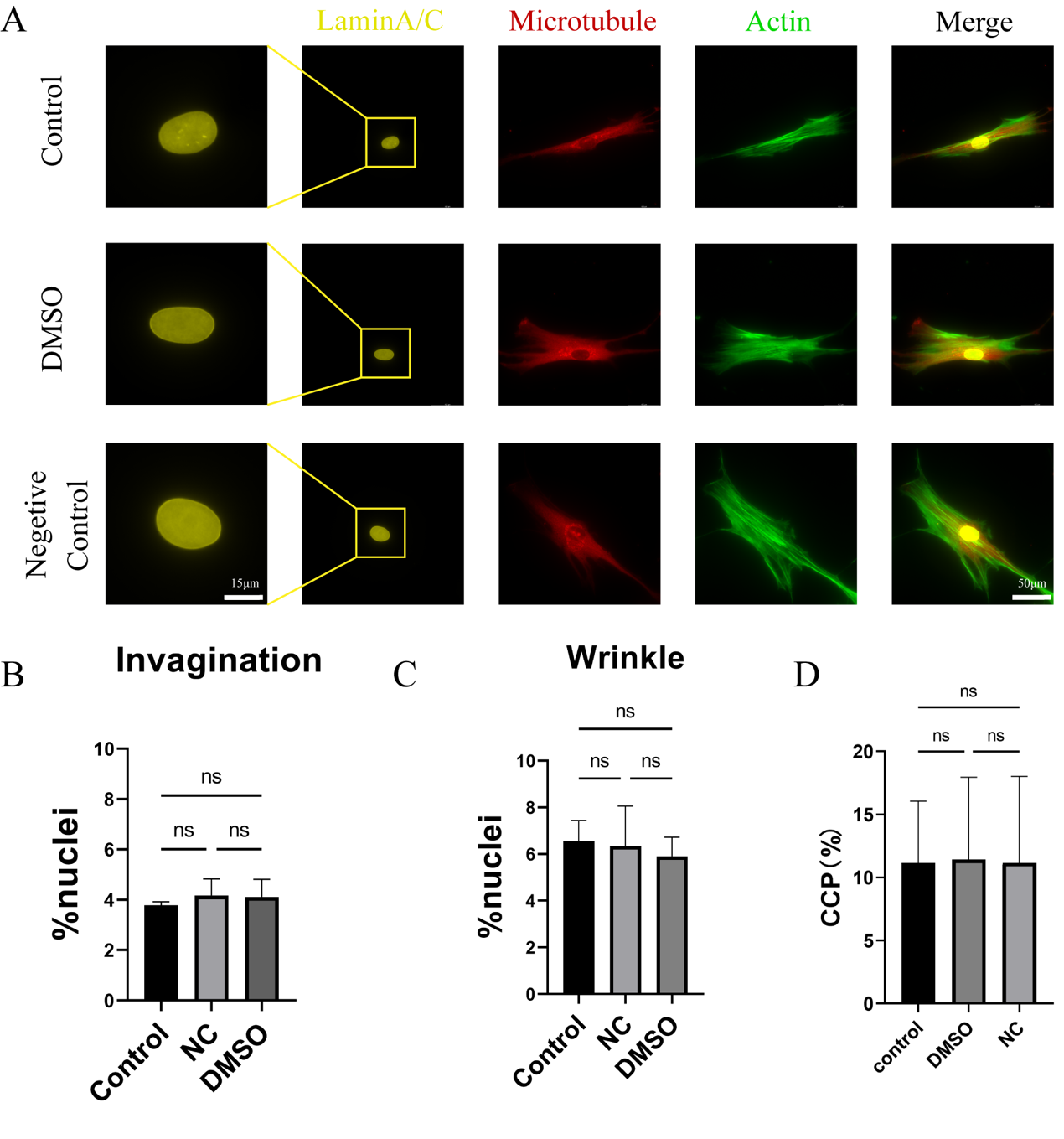


**Supplement Figure 1.** Dimethylsulfoxide (DMSO) and Negative control of siRNA did not affect cell states in the experimental conditions. A) Representative immunofluorescence images for lamin A/C, microtubule, and actin. B and C) The percentage of invaginated and wrinkle nucleus. D) The chromatin condensation parameter (CCP) was estimated by corresponding edge detection method (n = 15 three technical replicates).


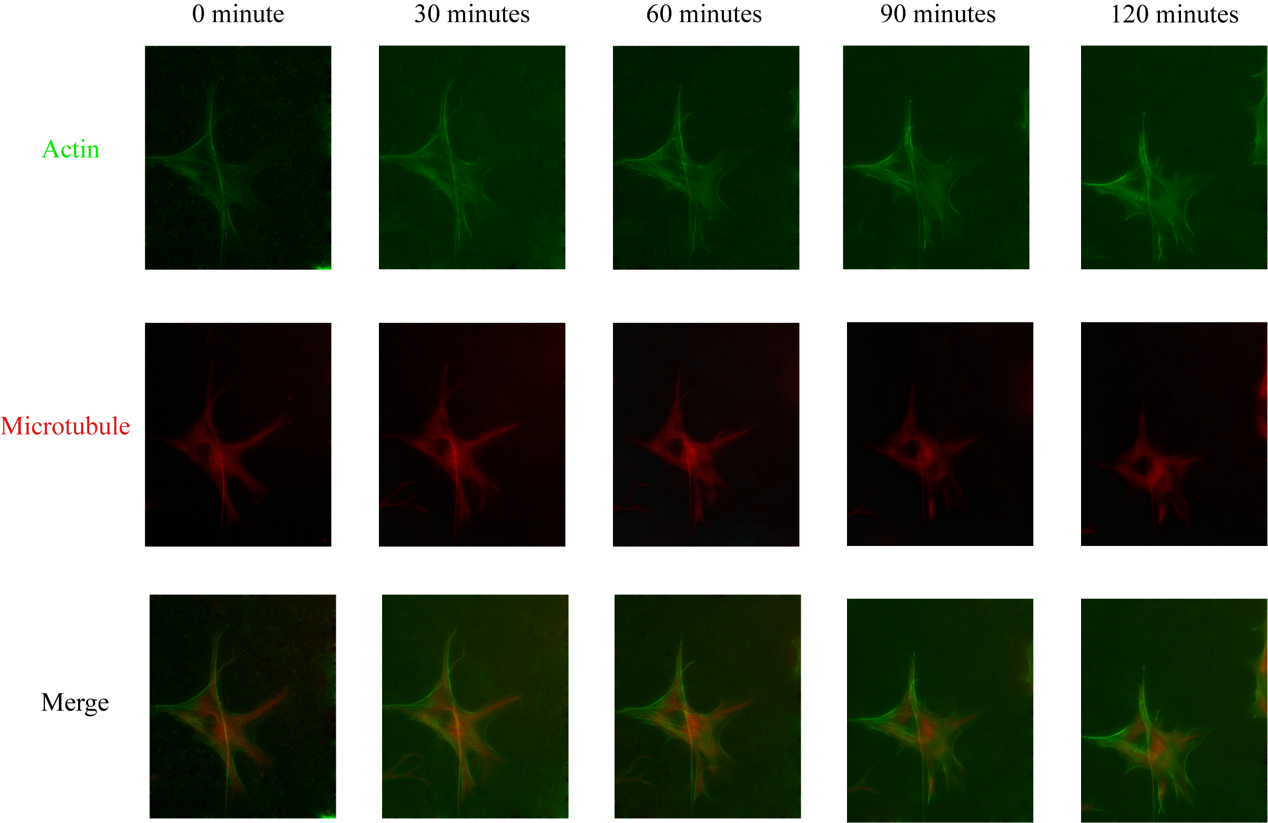


**Supplement Figure 2.** The time lapses images of the disruption of the actomyosin and microtubule in the cells treated with bleebistatin from Supplement Video 1-3. The actin filaments were gradually disordered after adding blebbistatin, which was accompanied by the microtubules gathering to the perinuclear area.


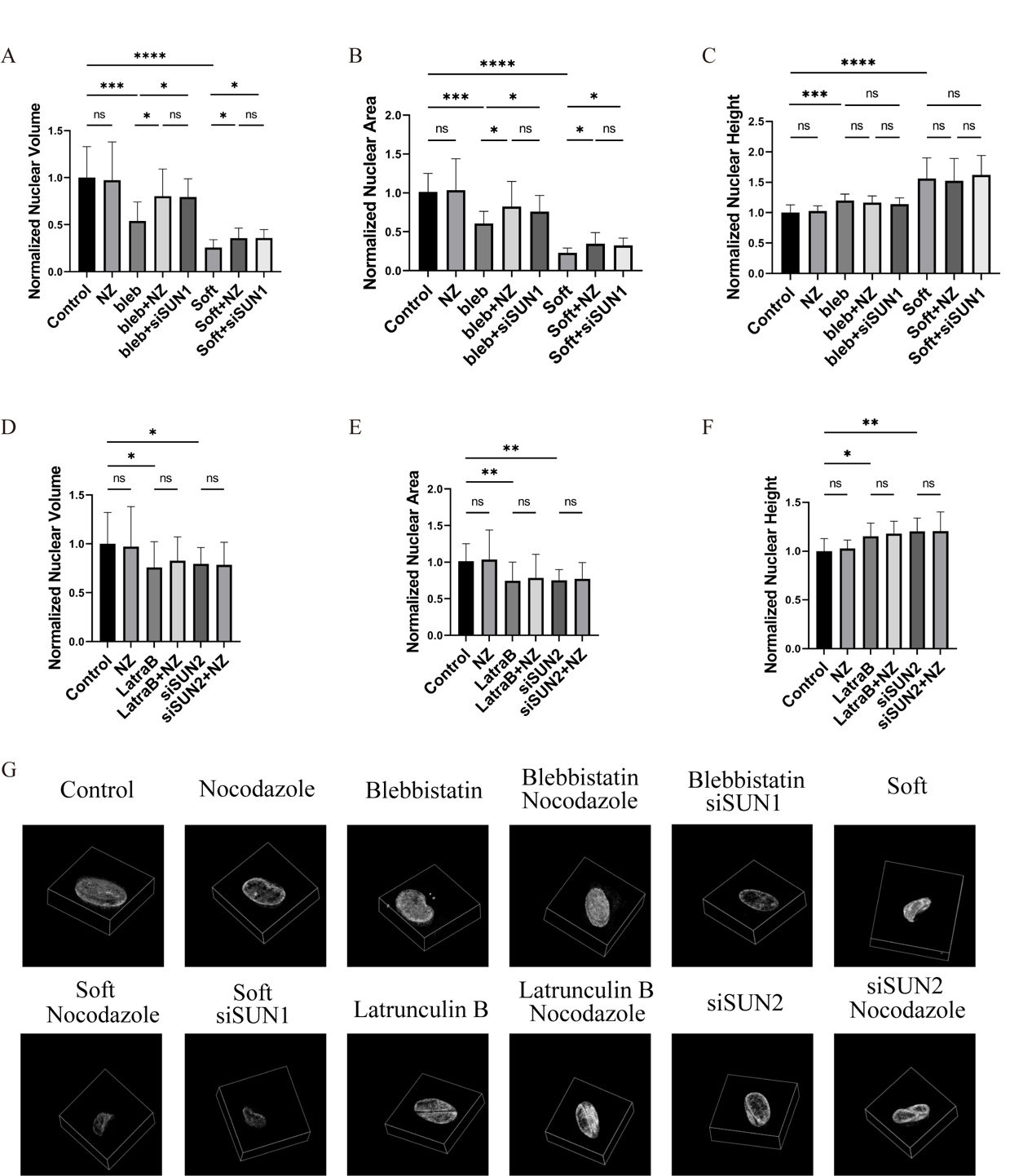


**Supplement Figure 3.** The nuclear volume, area, height (A-F, n = 15 three technical replicates), and the representative 3D images (G) of the cells in the representative groups.


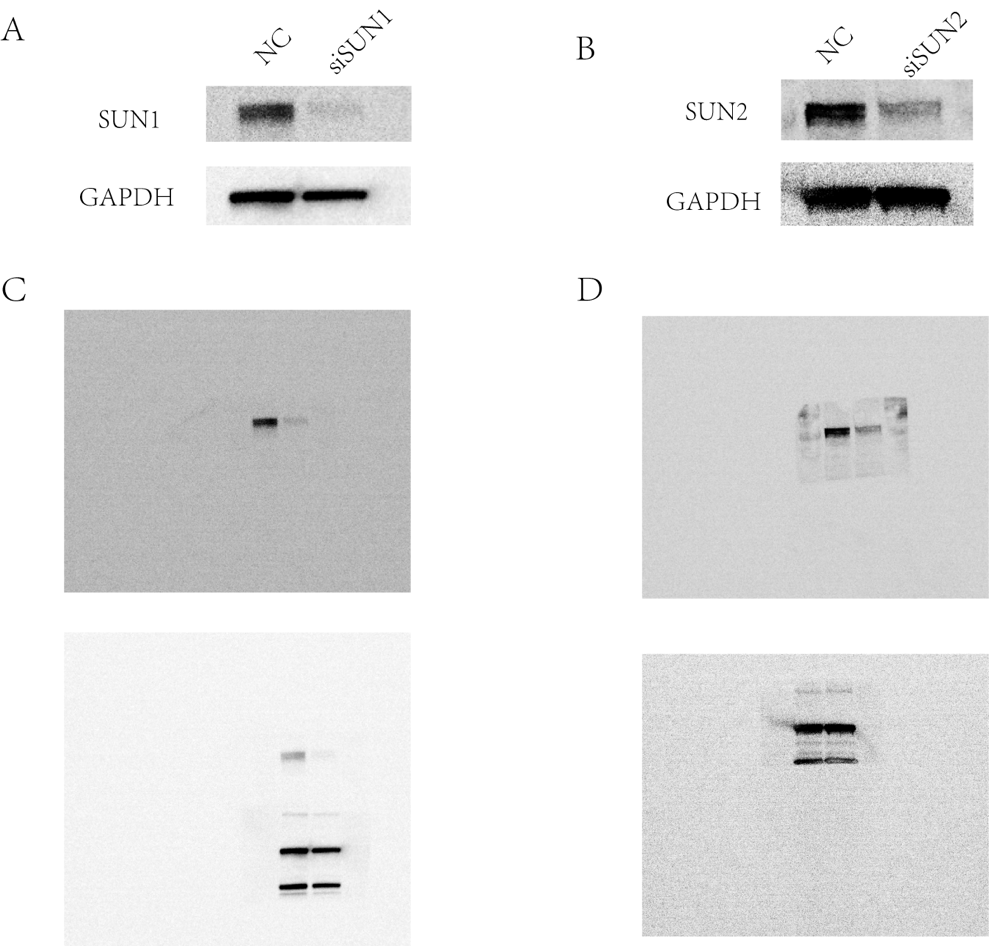


**Supplement Figure 4.** A and B) Western blotting shows comparable quantitative knockdown of SUN1 or SUN2 protein levels by siRNA as compared to cells transfected with negative control (NC) siRNA. C and D) The original pictures of western blot.


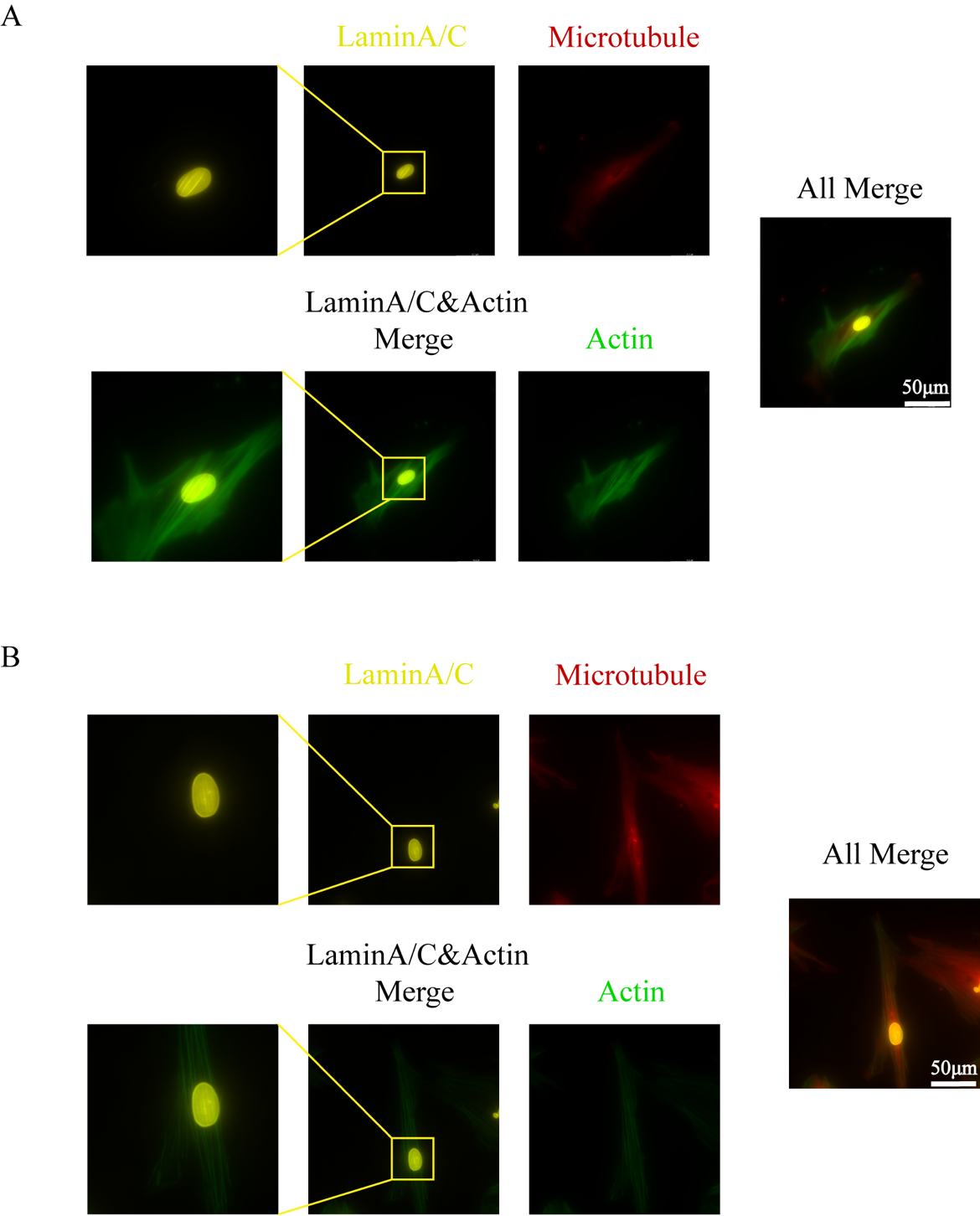


**Supplement Figure 5.** A) With actin cap inhibition by low concentration of latrunculin B or B) LINC disruption by SUN2 knockdown, many nuclei appeared wrinkles just under the remaining actomyosin fibers, which may be cause by the pressure from the actomyosin fibers.

**
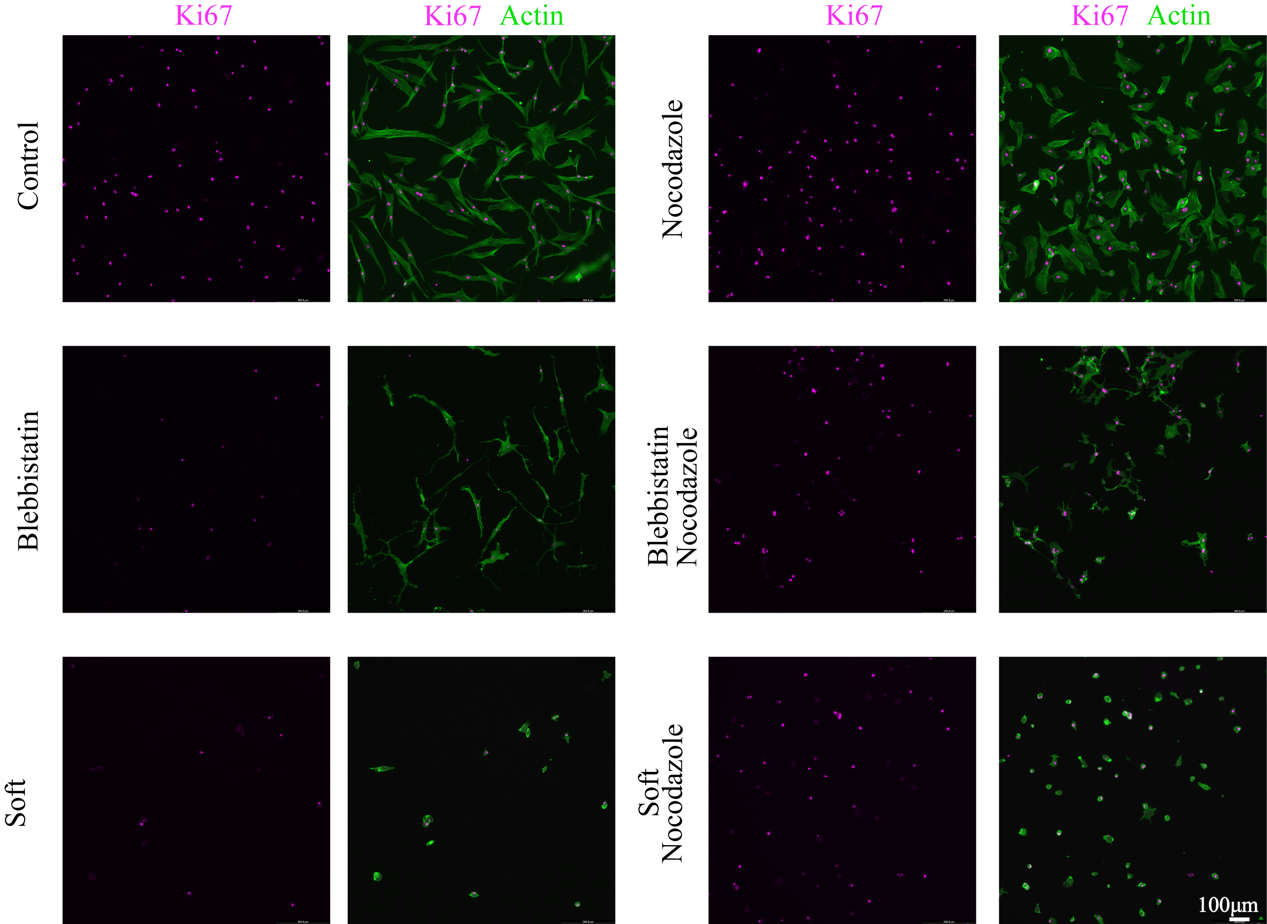
**

**Supplement Figure 6.** Enlarged representative immunofluorescence images for ki67 of the cells treated in shown conditions.

**Supplementary Table 1 | sequences of siRNA used in this study**

| siRNA | SENSE STRAND SEQUENCE |
| --- | --- |
| SUN1 | GGACGAGTCTTGGATTCGT |
| SUN2 | CCGTTACCTTAGAGCATGT |
